# Factors affecting height in children and adolescents with transfusion-dependent Thalassaemia – results from a Thalassaemia center in Malaysia

**DOI:** 10.1186/1687-9856-2013-S1-P61

**Published:** 2013-10-03

**Authors:** Tzer Hwu Ting, Bina Sharine Menon, Hishamshah Mohd Ibrahim

**Affiliations:** 1 Department of Paediatrics, University Putra Malaysia, Malaysia; 2 Haematology & Oncology Unit, Paediatrics Institute, Kuala Lumpur Hospital, Malaysia

## 

Thalassaemia, a common inherited haemoglobinopathy in Malaysia^1^, causes significant morbidity due to chronic anaemia and complications of iron excess from regular blood transfusion. Short stature is one of the commonest complications in transfusion-dependent thalassaemia with prevalence of 31% to 75% ^2,3^. Causes of growth impairment include iron overload, nutritional deficiency, GH-IGF1 axis dysregulation, abnormal puberty and desferrioxamine toxicity^4,5^.

We aim to determine the prevalence of short stature, defined as height z-score ≤ -2, and the factors affecting height in thalassaemia patients attending the Haematology clinic at a tertiary referral center. This observational cross-sectional study involved patients aged 3-19 years, needing blood transfusion at least every 8 weeks, and without co-morbidities or endocrinopathy which affect growth.

Weight & height were measured. Puberty status was clinically assessed. Body mass index (BMI) was calculated as a marker of nutritional status. Mean of ferritin levels measured over the preceding one year was determined as a marker of iron overload.

There were 52 boys (mean age 11.2y ± 4.8y, mean height z-score -1.67 ± 1.11, mean BMI z- score -0.87 ± 1.44, mean ferritin 3316 ± 2092 µg/L) and 47 girls ( mean age 11.8y ± 4.2y, mean height z-score -1.59 ± 0.97 , mean BMI z-score -0.83 ± 0.95, mean ferritin 3005 ± 2356 µg/L). Overall 35/99 (35%) were short while 64/99 (64%) had normal height. 19/52 (37%) boys and 16/47 (34%) girls were short. 16/19 (84%) short boys were aged ≥ 9 years, 15/16 (94%) short girls were aged ≥ 8 years. Only one boy (aged 15y) and one girl (aged 14y) had delayed puberty. They were both short with height z-score -3.41 and -2.26 respectively.

Overall there was a moderate positive correlation between height z-score and BMI z- score (Pearson r = 0.388, p=0.00). There was no significant correlation between height z score and mean ferritin level (Pearson r = -0.044, p= 0.666).

Significant proportion (35%) of our transfusion-dependent thalassaemia patients have short stature, majority of them in the pubertal age group. Height is significantly associated with nutritional status but not ferritin level. Improvement in nutrition may improve height in these patients.

